# Evidence for antimicrobial activity associated with common house spider silk

**DOI:** 10.1186/1756-0500-5-326

**Published:** 2012-06-25

**Authors:** Simon Wright, Sara L Goodacre

**Affiliations:** 1 School of Biologoy, University of Nottingham, University Park, NG7 2RD, Nottingham, UK

**Keywords:** Spider, Silk, Antimicrobial, Antibacterial, *Bacillus subtilis*, *Escherichia coli*, *Tegenaria domestica*

## Abstract

**Background:**

Spider silk is one of the most versatile materials in nature with great strength and flexibility. Native and synthetically produced silk has been used in a wide range of applications including the construction of artificial tendons and as substrates for human cell growth. In the literature there are anecdotal reports that suggest that native spider silk may also have antimicrobial properties.

**Findings:**

In this study we compared the growth of a Gram positive and a Gram negative bacterium in the presence and absence of silk produced by the common house spider *Tegenaria domestica*. We demonstrate that native web silk of *Tegenaria domestica* can inhibit the growth of the Gram positive bacterium, *Bacillus subtilis.* No significant inhibition of growth was detected against the Gram negative bacterium, *Escherichia coli.* The antimicrobial effect against *B. subtilis* appears to be short lived thus the active agent potentially acts in a bacteriostatic rather than bactericidal manner. Treatment of the silk with Proteinase K appears to reduce the ability to inhibit bacterial growth. This is consistent with the active agent including a protein element that is denatured or cleaved by treatment. *Tegenaria* silk does not appear to inhibit the growth of mammalian cells *in vitro* thus there is the potential for therapeutic applications.

## Background

Spiders use silk for a variety of different purposes such as web spinning, cocoon construction and as substrates upon which to deposit sperm. Silks vary in their mechanical properties such as in the degree of extensibility and maximum strength [1][2]. The range of physical properties observed likely reflects differences amongst silk types in the relative proportions and combinations of particular amino acids such as alanine, glycine and proline [3].

Spider silks can be grouped into five basic categories: dragline (also known as major ampullate), capture spiral, tubiliform, aciniform and minor-ampullate silks, each of which is used for a different purpose. In this study we examined the properties of combined samples of dragline and capture spiral silk from the agelenid spider *Tegenaria domestica* (Clerk 1757). This spider weaves funnel webs and is commonly found in leaf detritus or underneath rocks [4]. It uses dragline silk for creating the web’s outer rim and spokes, and also for the lifeline that attaches the spider to a surface. Capture spiral silk is used for the capture lines on the web. As its name suggests, it is used for capturing prey as they collide with the web. In order to do this it stretches so as to prevent breakage by absorbing the impact energy. Capture spiral silk is also sticky. Glycoproteins covering the surface are important in determining both the stickiness and stretchiness of the silk [5].

There are records indicating that spider silk has historically been used by humans for a variety of purposes. Peoples of the Carpathian Mountains are reported as using sections of the tubular shaped webs of *Atypus* spiders as topical bandages to heal wounds. This was believed to be beneficial due to the antiseptic properties of the spider silk [6]. Few studies, however, appear to have investigated this property in detail. In this study we challenged two types of bacterial cultures, Gram negative *E. coli* and Gram positive *B. subtilis,* with naturally spun spider silk using an *in vitro* assay. We examined the growth response of bacteria to native silk and to silk that had been chemically treated in order to investigate what the active antimicrobial agent might be. We also exposed mammalian cell cultures to natural spider silk in order to examine whether silk appears to be tolerated by these types of cell, a factor that is important in assessing the potential for subsequent therapeutic applications in the treatment of human disease.

## Methods

### **Tests for antimicrobial activity**

To test for antimicrobial activity of spider silk we incubated bacterial cultures in the presence and absence of silk and measured bacterial growth using photospectrometry. 10 ml of liquid Luria broth in a sterile 20 ml universal tube was inoculated with 50 μl overnight culture of either *E. coli* or *B. subtilis.* Silk was collected by running a sterile pipette through the web to collect a sheet of approximately 7 mm^2^. Both the silk and the sterile pipette with which the silk was collected were submerged in the mix of the liquid broth and microbe. The tubes were placed on a shaker rotating at 150 rpm and heated to 27°C. After incubation, 2mls of the sample was pipetted into a cuvette which was placed in a photo-spectrometer (Biochrom Libra S6) and the absorbance read at 660 nm. Light absorbance is expected to be proportional to bacterial density in the medium with absorbance increasing with microbial density. All silk samples were incubated for 24 hours with a subset incubated for a further 24 hours. A series of tests using freshly spun silk from a single UK *Tegenaria* male were carried out over the space of a few weeks. Each experimental culture containing silk was paired with a control culture inoculated at the same time with bacteria but no silk.

Statistical analyses of light absorbance data were made using both the sign test and the paired samples t-test. The *a priori* prediction that silk reduces growth was used and thus the results are reported as 1-tailed.

To further characterise the active agents on the spider silk that inhibit the growth of microbes, silk was subjected to a range of different treatments; (i) exposing to ultraviolet light (254 nm) for 20 minutes; (ii) soaking in sterile distilled water for 1–2 hours before use and (iii) incubating with Proteinase K for 1–2 hours before use. Soaking in Proteinase K was used to establish whether the agent was behaving as if it was a protein *i. e.* being destroyed or denatured under the conditions applied. Exposure to UV light at 254 nm is expected to damage DNA and thus affect microorganisms that are already present on the silk sample, but not necessarily to denature proteins. Soaking in water was used to see if any active agent was present on the silk surface and soluble.

### **Mammalian cell line**

The effects of spider silk on mammalian cell lines were investigated by incubating silk with Jurkat cells. Jurkat cells were removed from liquid nitrogen storage and thawed in a water bath at 37°C. After heating for around 10 minutes, approximately 3x10^5^ cells were added to 5 ml of mammalian cell growth medium and incubated at 37°C in the presence of 5% CO_2_. They were then left to grow for two days, during which time the pH of the culture was measured daily, before being removed and the number of cells calculated by taking 20 μl of cell suspension, staining cells with an equal volume of Trypan Blue dye and counting the number of cells using a haemocytometer.

Mammalian cells are highly susceptible to bacterial infections therefore the spider silk used was subjected to 20 minutes of UV irradiation before the experiment by placing for 20 minutes beneath a UV source in a laminar flow hood (254 nm, 15 watts). Exposure at this wavelength is expected to reduce or eliminate any bacteria that are present on the silk and had been shown by preliminary trials not to eliminate the antimicrobial activity of the silk.

## Results

### **Tests for antimicrobial activity**

A total of 18 paired trials were carried out testing the effect of *T. domestica* silk on *B. subtilis* growth (Table 1). After 24 hours a significant proportion of trials (14/18) showed less *B. subtilis* growth when in the presence of *Tegenaria* silk (mean absorbance: *B. subtilis* alone = 0.76, *B. subtilis* plus silk = 0.64, sign test +14, -4, *P* = 0.015). Paired samples t-tests, which take the magnitude of the difference between silk and control samples into account also support the conclusion that bacterial growth is less when in the presence of silk (t = 2.052, 17 d.f., *P* = 0.028).

**Table 1:** **Table of absorbance readings of growth of*B. subtilis*when exposed to the silk of*T. domestica***

**Trial No.**	**Control absorbance reading**	**Silk sample reading**	**Inhibition %**	**Sign test**
1	0.34	0.315	7.352941	+
2	0.34	0.28	17.64706	+
3	0.32	0.28	12.5	+
4	0.57	0.54	5.263158	+
5	0.5	0.54	−8	-
6	0.585	0.582	0.512821	+
7	0.575	0.57	0.869565	+
8	0.63	0.44	30.15873	+
9	1.22	0.57	53.27869	+
10	1.29	0.42	67.44186	+
11	0.61	0.56	8.196721	+
12	0.58	0.53	8.62069	+
13	0.6	0.5	16.66667	+
14	0.71	0.77	−8.4507	-
15	0.64	0.55	14.0625	+
16	1.03	1.06	−2.91262	-
17	0.95	0.96	−1.05263	-
18	2.1	2	4.761905	+

8 trials were selected at random to be incubated for a further 24 hours. The mean absorbance of *B. subtilis* was less when incubated with silk (mean absorbance with silk = 0.54, without silk = 0.56) but the difference was not significant (paired samples t-test *P* = 0.36). 4 trials showed less bacterial growth in the presence of silk and 4 showed more growth (sign test *P* = 1).

25 paired trials were carried out to test the effect of *Tegenaria* silk on *E. coli* growth (Table 2). 15 of these trials showed lower growth in the presence of silk, 7 showed more growth and 3 showed equal growth, but the difference between silk and control samples was not significant (mean absorbance: *E. coli* alone = 0.86, *E. coli* plus silk = 0.88, sign test +15, -7 *P* = 0.07, t-test *P* = 0.8). Of the 8 trials selected at random to be incubated for a further 24 hours, 5 showed less growth in the presence of silk but the difference remained non-significant (sign test *P* = 0.36, t-test *P* *=* 0.81).

**Table 2:** **Table of absorbance readings of growth of*E.coli*when exposed to the silk of*T. domestica***

**Trial No.**	**Control absorbance reading**	**Silk sample reading**	**Inhibition %**	**Sign test**
1	0.06	0.055	8.333333	+
2	0.15	0.07	53.33333	+
3	0.11	0.065	40.90909	+
4	0.13	0.08	38.46154	+
5	0.15	0.11	26.66667	+
6	0.07	0.065	7.142857	+
7	0.33	0.33	0	
8	0.43	0.4	6.976744	+
9	1.244	1.22	1.92926	+
10	0.33	0.35	−6.06061	-
11	0.33	0.33	0	
12	1.244	1.15	7.55627	+
13	1.244	1.19	4.340836	+
14	1.25	1.21	3.2	+
15	0.74	0.77	−4.05405	-
16	1.08	1.03	4.62963	+
17	0.88	1.15	−30.6818	-
18	0.76	1.26	−65.7895	-
19	0.89	1.18	−32.5843	-
20	0.94	1	−6.38298	-
21	0.58	0.57	1.724138	+
22	1.03	1.01	1.941748	+
23	2.5	2.5	0	
24	2.41	2.42	−0.41494	-
25	2.5	2.45	2	+

### **Silk treatment with ultraviolet light, soaking in water and Proteinase K and incubation with mammalian cells**

5 paired trials carried out using silk exposed to ultraviolet light (Table 3) showed that the silk still appeared to retain antimicrobial activity against *B. subtilis* (mean absorbance for control = 0.51, with silk = 0.48, sign test *P* = 0.03, paired samples t-test t = 3.59, *P* = 0.016).

**Table 3:** **Table of absorbance readings of growth of*B. subtilis*when exposed to the ultraviolet-light treated silk of*T. domestica***

**Trial No.**	**Control absorbance reading**	**Silk sample reading**	**Inhibition %**	**Sign test**
1	0.32	0.26	18.75	+
2	0.54	0.53	.93	+
3	0.39	0.37	5.13	+
4	0.58	0.55	5.17	+
5	0.71	0.68	4.23	+

In contrast, silk that had been soaked in water or incubated with Proteinase K no longer appeared to have any antimicrobial activity (Tables 4, 5). In tests using silk that had been soaked in water only 3 out of 7 trials showed less growth in the presence of silk (mean absorbance for *B. subtilis* alone = 0.61, for *B. subtilis* plus water-soaked silk = 0.68). In tests using silk that had been soaked in Proteinase K only 1 out of 4 trials showed less growth in the presence of silk (mean absorbance for *B. subtilis* alone = 0.67, for *B. subtilis* plus Proteinase K-soaked silk = 0.76).

**Table 4:** **Table of absorbance readings of growth of*B. subtilis*when exposed to the water soaked silk of*T. domestica***

**Trial No.**	**Control absorbance reading**	**Silk sample reading**	**Inhibition %**	**Sign test**
1	0.61	0.55	9.84	+
2	0.54	1.00	−86.92	-
3	0.71	0.65	8.45	+
4	1.10	1.06	3.64	+
5	0.42	0.53	−26.19	-
6	0.42	0.49	−16.67	-
7	0.49	0.51	−4.08	-

**Table 5:** **Table of absorbance readings of growth of*B. subtilis*when exposed to the Proteinase-K soaked silk of*T. domestica***

**Trial No.**	**Control absorbance reading**	**Silk sample reading**	**Inhibition %**	**Sign test**
1	0.49	0.50	−2.04	-
2	0.50	1.29	−158.00	-
3	0.55	0.51	7.27	+
4	1.16	1.20	−3.45	-

4 paired trials were carried out incubating UV treated spider silk with mammalian Jurkat cels (Table 6). Treatment with UV had already been shown not to remove antimicrobial activity and was performed in this case to reduce/eliminate bacteria that were naturally contaminating the silk and whose growth might influence growth of mammalian cells. Two trials had greater numbers of cells in the control and two had greater numbers in the sample containing silk but there was no significant difference between the control and silk-containing samples (mean cell count of control = 0.67, mean cell count of silk samples = 0.67). There was no evidence to suggest that the presence of silk alters the pH of the *in vitro* cell culture or Luria broth media or sterile distilled water (zero difference between samples with and without silk was detected 24 hours and 1 week after the start of incubation).

**Table 6:** **Table of Jurkat cell counts per ml and pH when exposed to silk of*T. domestica***

**Trial No.**	**Number of million control cells per ml**	**Number of million silk cells per ml**	**Control pH 1 day**	**Silk pH 1 day**	**Control pH 1 week**	**Silk pH 1 week**
1	1.12	1.08	6.4	7	7.4	6.5
2	0.72	0.54	6.7	6.7	6.7	6.7
3	0.44	0.52	7.2	6.7	7.2	6.7
4	0.4	0.54	7.2	7.2	7.2	7.2

## Discussion

Natural materials have historically been a source of novel antimicrobial agents. It is estimated that from the mid-1980s to the mid-1990s 78% of new drugs came from biological sources such as soil [7]. In this study we demonstrate that silk from the common house spider, *T. domestica,* appears to reduce the growth of *B. subtilis* under laboratory conditions. The effect appears to decrease over time with the significant effect detected at 24 but not 48 hours after the start of the experiment. A possible explanation for the apparent reduced activity with time is that the active agent is bacteriostatic in action, slowing the growth of the bacteria rather than killing them. Bacteriostatic modes of activity against *B. subtilis* are already documented in the literature [8-10] and it is conceivable that the active agent in silk also operates in this manner.

Our study of *T. domestica* silk shows that the antimicrobial activity against *B. subtilis* is reduced or negated after digestion with Proteinase K. The reduction of antimicrobial properties after treatment with Proteinase K indicates that at least one protein may be involved. Spider silk is known to be coated in glycoproteins [11] approximately 150-250 nm thick. It is possible the antimicrobial property of the spider silk comes from these glycoproteins. Exposure to ultraviolet light, which is expected to damage the DNA of microorganisms [12] appeared not to eliminate the activity. A non-spider origin of the antibacterial agent, such as secretion of proteins toxic to *B. subtilis* by bacteria already present on the silk, thus also appears unlikely to fully explain the inhibition of *B. subtilis* growth.

A similar (but non-significant) trend of reduced *E. coli* growth in the presence of *Tegenaria* silk was also observed*.* This may reflect the fact that our experimental set up was not sensitive enough to detect activity against this microbe or it could reflect real differences between activity against the two types of bacteria, a characteristic common to many other antimicrobial agents, which may have narrow spectra of activity [13-15]. In this context it is interesting to note that *B. subtilis,* unlike *E. coli,* is a soil bacterium [16]. We cannot completely exclude the possibility that activity against *E. coli* remains undetected in our experiment but we note that silk regularly comes into contact with soil and is much more likely to encounter *B. subtilis* than *E. coli* and thus have evolved resistance to it. It should be noted however that only two species of bacteria were tested in this experiment and thus predictions about the effect of spider silk on other species of gram positive or negative bacteria cannot be inferred from the data we have gathered.

Our initial tests of the response of mammalian cell lines cultured *in vitro* to *Tegenaria* silk indicate that the silk itself and any associated protein or chemical agents are not toxic, potentially paving the way for therapeutic applications*.* Modern uses of spider silk already involve using silk from spiders such as *Nephila clavipes* to help in mammalian neuronal regeneration [17]. *Nephila* silk in this case did not appear to provoke an auto immune response. This is an important factor to consider because if a material is to be used therapeutically then it is important that the material is neither toxic nor swiftly destroyed by the recipient’s immune system.

Other recent biotechnological applications of spider silk includes the use of recombinant spider silk particles as drug delivery vehicles [18]. Spider silk has also been suggested to be used as a load bearing biomaterial by Brown [19] because of its biocompatibility, and strength and toughness. Other potential uses have been proposed with applications ranging from artificial tendons [20] to rust free panels having been suggested [21]. Spider silk has been suggested as a suitable replacement material for many existing products such as clothing, ropes, seat belts, body armour, parachutes and biodegradable bottles, all of which could show both cost and environmental benefits if made from spider silk rather than current manmade materials [21]. We suggest that an added benefit to these sorts of applied uses is the potential for the products to have a level of intrinsic antimicrobial activity.

Our experiment demonstrates that an antimicrobial effect exists in silk in its native form, a property that may have evolved in order that silk can resist microbial decomposition. Reduced decomposition might be advantageous if it decreases the energetic costs of web maintenance and/or the level of harmful microbes to which a spider is exposed. The effect might be even more strongly selected for in silks that protect developing eggs, which might be particularly adapted to resist decomposition or destruction by predators, parasites or fluctuations in external abiotic conditions [22]. Reducing the bacteria present on the silk could also make the web harder to detect by prey by visual or olfactory signals. In addition it has been observed that the presence of aciniform silk leads to a delay in prey decomposition [23] reducing the exposure to microbes and increasing the amount of substance ingested. It remains to be seen whether antimicrobial activity is observed in other silks from a wider range of species and if so, whether the traits are homologous or have evolved independently as a response to similar microbial challenges in their environment.

## Competing interests

The authors declare that they have no competing interests.

## Authors' contributions

SW conceived of the study, carried out the data collection and analysis and drafted the manuscript. SLG participated in the design of the study and helped draft the manuscript. Both authors read and approved the final manuscript.

## Authors’ information

SLG is a RCUK Academic Fellow, in the Faculty of Medicine & Health Sciences at the University of Nottingham and runs the 'SpiderLab' at Nottingham, working on a range of evolutionary, population and conservation genetic studies using spiders as model systems. SW at the time of the research was a Masters of Research student at the University of Nottingham supervised by SLG.
